# Biogrout: A Novel Binding Material for Soil Improvement and Concrete Repair

**DOI:** 10.3389/fmicb.2016.00314

**Published:** 2016-03-17

**Authors:** Varenyam Achal, Satoru Kawasaki

**Affiliations:** ^1^Shanghai Key Lab for Urban Ecological Processes and Eco-Restoration, School of Ecological and Environmental Sciences, East China Normal UniversityShanghai, China; ^2^Faculty of Engineering, Hokkaido UniversitySapporo, Japan

**Keywords:** grout, biogrout, calcite, soil improvement, concrete

## Grout

Grout is a binding agent or binder often used with building materials to improve their durability functions. It may be organic or inorganic material used for sealing or consolidation of cracks, pores, or voids in order to improve the mechanical properties and impermeability of a particular system, whether soil, cementitious or of other building materials. Grouts act as dispersion agents where dispersed particles form a network responsible for the effectiveness of grouting properties. Chemical grouts are the most common and well-known grouting materials used for sealing sand, soil, or other matrices. However, this is an expensive technique and it also affects the environment because chemical grouts based on acrylamides, lignosulfonates, and polyurethane are toxic and environmentally harmful. Thus, it is necessary to find new grouting materials and methods of injection. It is important to choose grouting materials that are soft and fine enough to penetrate into the discontinuities in voids, cracks, or cavities to fill them (Bras et al., 2013).

## Biogrout

On the other hand, various minerals including calcium carbonate, calcium phosphate, calcium oxalate, silicate, and iron oxide are precipitated in nature by living organisms (Mann, 1993). These biominerals are promising engineering materials because they provide adequate strength and low environmental impact. In order to reduce environmental impact and cost in the future, a novel grouting method that utilizes calcium carbonate precipitated by bacterial cells has been examined recently. Such grout is commonly called “biogrout,” a product of biomineralization which has the potential to be a better alternate grouting material. A novel grout should have a self-leveling behavior in addition to a high compressive strength and low shrinkage, be easy to inject, and have good durability (Bras et al., 2013); this can be achieved using biogrout. Furthermore, as the microbial reaction in biogrout is slower than the chemical reaction in chemical grouts, this reduces the solidification speed, which allows the biogrout to spread through a greater volume of soil or other matrices (Akiyama and Kawasaki, 2012a,b).

Biogrouting technology is similar to chemical grouting where the depth of penetration depends on the size of bacteria used, in addition to the optimal conditions for bacterial activity such as pH, salinity, oxidation-reduction potential, concentrations of nutrients, and content of water. Biogrout is calculated to be much cheaper than chemical grouting techniques. Raw materials increase the cost in chemical grouts ranging from $2 to 72 per m^3^ of soil, compared to biogrouts in the range of $0.5 to 9.0 per m^3^ of soil, in cases where waste materials are used as the source of carbon for bacterial growth (Ivanov and Chu, 2008).

## Application of biogrout

Biogrout was successfully used to control ground permeability and to reinforce the ground with bacterially produced cementing materials (Whiffin et al., 2007; Ivanov and Chu, 2008; van Paassen et al., 2009; DeJong et al., 2010; Harkes et al., 2010). Most studies on biogrout development are mainly based on microbial induced calcite precipitation (MICP), a process involving ureolytic bacteria and urea as a substrate. During this process, bacterial urease hydrolyzes urea that increases the pH by producing ammonium ions and generates CO_2_. Furthermore, CO_2_ dissolution takes place and carbonate ions combine with calcium ions present in the proximal environment or on the cell wall of bacterial cells to precipitate calcium carbonate crystals (Li et al., 2015). These crystals create bridges between the sand grains, by which the soil is strengthened or cracks are sealed in the concrete. The precipitation of the solid calcium carbonate decreases the porosity and the permeability (van Wijngaarden et al., 2011). Furthermore, compared to cementitious soil or water saturated matrices, non-water saturated sandy soils such as sand dykes, road or train embankments, and sand dunes are hard to grout due to the decreased control of flow that can be exercised in the non-saturated soil environment. However, biogrout has resulted in homogeneous cementation over the entire length of a 1-m sand column, where calcite crystals provided strength related to the pore water content of the continuously drained column with less water content enabling more efficient strength formation (Cheng and Cord-Ruwisch, 2012). Scaled up experiments provided technical feasibility of BioGrout as a ground improvement method under real life conditions and with practical techniques, both as a single point injection or over a horizontal distance using screens of injection and extraction wells, where unconfined compressive strength (UCS) varied considerably from 0 to 12 MPa (van Paassen et al., 2009). Such results open a way for biogrout to be applied in an open system *in situ*. Biogrout also plays an important role in sealing cracks in concrete, as bacterial cells grow in the presence of moisture, water, or oxygen, followed by urease production and ultimately calcite precipitation that seals cracks (Achal and Mukherjee, 2015).

Urease-based biogrout is commonly used by various researchers to improve mechanical properties, decrease the permeability of porous materials, repair cementitious materials, and modify the properties of soil, or sand (Whiffin et al., 2007; DeJong et al., 2010; Rong et al., 2012); however, this process suffers from excessive ammonia production. In order to solve this problem, recently biogrout was produced by an asparaginase-based MICP process, where MICP followed ammonification i.e., the process of amino acid deamination after hydrolysis of protein by heterotrophic bacteria as the pH-increasing reaction (Galloway, 2005). Biogrout based on asparaginase resulted in UCS of 980 kPa in sand compared to that of 1002 kPa due to urease-based MICP process (Li et al., 2015).

## Advantage of biogrout

The results suggest that there are possibilities to reduce the drawbacks of biogrout production such as reducing the effect of ammonia on the environment without compromising the mechanical and impermeable properties of biogrout in sand. The developed biogrouts have been shown to be economical, environmentally friendly, and that the rheological characteristics can be appropriate for injection applications to consolidate cracks in concrete and for soil improvement. We believe that biogrout will have a wider acceptance in future research and will attract more researchers to work on it.

## Author contributions

All authors listed, have made substantial, direct and intellectual contribution to the work, and approved it for publication.

### Conflict of interest statement

The authors declare that the research was conducted in the absence of any commercial or financial relationships that could be construed as a potential conflict of interest.
